# Responses of microbial community structure in turbot (*Scophthalmus maximus*) larval intestine to the regulation of probiotic introduced through live feed

**DOI:** 10.1371/journal.pone.0216590

**Published:** 2019-05-08

**Authors:** Yan Jiang, Yingeng Wang, Zheng Zhang, Meijie Liao, Bin Li, Xiaojun Rong, Guiping Chen

**Affiliations:** 1 Yellow Sea Fisheries Research Institute, Chinese Academy of Fishery Sciences, Qingdao, China; 2 Laboratory for Marine Fisheries Science and Food Production Processes, Pilot National Laboratory for Marine Science and Technology (Qingdao), Qingdao, China; Institut National de la Recherche Agronomique, FRANCE

## Abstract

Various bacteria that adhere to the gut are important for the health of fish. Regulating the microbial community in a desirable direction may be beneficial in aquaculture for preventing and controlling the diseases caused by pathogenic microbes. In this study, we investigated the changes in the microflora in the intestinal tracts of turbot (*Scophthalmus maximus*) larvae after introducing a probiotic (*Bacillus amyloliquefaciens*) after the first feed. *B*. *amyloliquefaciens* was added as part of a nutrient enrichment system in live feed (*Branchionus plicatilis* or *Artemia sinica*), so it passed into the intestinal tracts of the newly hatched turbot larvae. The turbot larvae were fed on live feed containing *B*. *amyloliquefaciens* in the experimental group, whereas live feed without the probiotic was provided to larvae in the control group. The total bacterial genomic DNA in the larval guts was extracted and sequenced with an Illumina HiSeq PE250 system. According to the sequencing results, the abundances of microbial species and the microflora diversity were lower in the intestines in the experimental group than the control. Throughout development, the microflora structure in the intestines was mainly constructed before the first feed and the composition of the dominant operational taxonomic units (OTUs) was stable, where the abundances of OTU8, OTU124, OTU150, OTU107, and OTU17 were always high. Compared with the control, the structures of the microflora in the intestines were similar on different days during the development and the growth of larvae in the experimental group. However, the similarity of the microflora structure between different treatments was low on the same day. Furthermore, the mean proportion of common OTUs was only 74.7% in different treatments on each day, which indicates that the introduction of *B*. *amyloliquefaciens* in the live feed changed the microflora structure in the intestine. During the early development stage (days 3–30), the average abundance of *Pseudomonas* was reduced by 0.8% whereas that of *Lactococcus* increased by 3.5% in the experimental group. *Pseudomonas* spp. are considered potentially pathogenic bacteria but there is no direct evidence for the pathogenicity of *Lactococcus* in turbot. Moreover, several *Lactococcus* species are regarded as probiotics in aquaculture. Therefore, the use of *B*. *amyloliquefaciens* could be beneficial for optimizing the microbial community structure in the intestines of turbot larvae, which may explain the probiotic effect of *B*. *amyloliquefaciens*. This study provides a theoretical basis for the biological regulation of the microflora structure in the intestinal tract during turbot breeding.

## Introduction

Aquaculture is considered to be a very important food production system and it is one of the fastest growing industries that can meet the increasing demand for high quality animal protein. Turbot (*Scophthalmus maximus*) is one of the most economically important species throughout the world, where the total production reached 77,117 tonnes in 2012, although small gains were obtained in 2013 [1]. Bacterial diseases are among the most significant problems that affect aquaculture. In particular, bacterial diseases cause a wide range of severe infections that may lead to death, where the growth and propagation of pathogenic bacteria are fairly rapid. In the larval stages [2], diseases such as tenacibaculosis and vibriosis caused by bacterial pathogens [3, 4] may cause the death of all infected individuals in turbot farming, and thus the aquaculture industry will be severely affected with great economic losses.

Traditionally, the control of pathogens has been based on antibiotics, although their effectiveness is minimal or limited. In fact, antibiotics do not effectively control the growth and reproduction of undesirable microbes, and they may lead to undesirable changes in pathogenic bacteria such as the emergence of antibiotic resistance [5–7]. Other antimicrobial strategies such as vaccination have been proposed for disease control and applied quite successfully in aquaculture [8–11]. However, there are no appropriate vaccines for some of the diseases caused by pathogenic bacteria, such as *Vibrio splendidus* [12]. A probiotic is defined as: “live microorganisms that, when administered in adequate amounts, confer a health benefit on the host” [13], and probiotics may be suitable for improving disease resistance and the immune response, as well as enhancing the supply of nutrients [14–20]. Lactic acid bacteria such as *Enterococcus*, *Lactobacillus*, *Leuconostoc*, *Carnobacterium*, and *Lactococcus* [2, 17, 21], and *Bacillus* [22–25] are generally considered to be probiotics in aquaculture.

The intestinal tract is the most important part of the digestive system and it is regarded as an indispensable metabolic organ. Microbes can successfully colonize the intestinal tract via competition and adhere to its surfaces. The balance of the microbial community in the gut has important beneficial effects on the host’s health. In particular, various bacteria that adhere to the surfaces of the gut can affect the absorption of nutrients and intestinal immune responses [26–29].

*Bacillus amyloliquefaciens* is considered a potential probiotic for use in aquaculture because it can protect aquatic animals from *Edwardsiella tarda*, *Aeromonas hydrophila*, *Vibrio parahaemolyticus*, and *Vibrio harveyi* [30, 31]. In this study, we added *B*. *amyloliquefaciens* to culture systems of *Branchionus plicatilis* and *Artemia sinica* during nutrient enrichment, and they were ingested by turbot larvae. The microflora present in the guts of the turbot larvae were then analyzed to assess the effects of *B*. *amyloliquefaciens* on the microbial community structure in the intestinal tract. Our findings provide insights into the potential application of probiotics during turbot farming.

## Materials and methods

### Culturing of probiotic bacteria

*Bacillus amyloliquefaciens* was stored in the laboratory after being isolated from the intestinal tract of healthy juvenile turbot by Fan [32]. In her study, the antagonism tests showed that *B*. *amyloliquefaciens* can inhibit the growth and reproduction of the common pathogens of turbot, such as *Vibrio carchariae*, *Vibrio scophthalmi*, and *Vibrio anguillarum*. Moreover, the study indicated that no pathological consequences or death were detected according to tissue analysis and clinical observation when the juvenile turbot were fed on a diet containing *B*. *amyloliquefaciens* at 10^9^ cfu g^–1^, and the addition of this strain could help for the health and growth of juvenile turbot.

*Bacillus amyloliquefaciens* was cultured in tryptic soy broth medium at 30°C for 1 day. We added 2% salt to the tryptic soy broth medium during the culture of the probiotic. Single colonies were isolated from solid medium and added to the liquid medium. The inoculated liquid medium was then incubated at 30°C in a rotary shaker at 180rpm for 10hrs. The concentration of *B*. *amyloliquefaciens* in suspension was 10^9^ cfu mL^–1^ according to the viable count on solid medium, which was used in nutrient enrichment of the live feed.

### Preparation of live feed

The live feed contained *B*. *plicatilis* and *A*. *sinica* during the turbot rearing period. Suspensions of *B*. *amyloliquefaciens* were added to the culture system of *B*. *plicatilis* or *A*. *sinica* in the nutrient enrichment process. This process continued for 6 h, and *B*. *plicatilis* or *A*. *sinica* were then collected to feed to the turbot larvae. The initial concentration of *B*. *amyloliquefaciens* was 10^6^ to 10^7^ cfu mL^–1^ in the culture system of *B*. *plicatilis* or *A*. *sinica*. In order to confirm the survival rate and quality, the nutrient enrichment of *B*. *plicatilis* or *A*. *sinica* was also performed without *B*. *amyloliquefaciens* according to the turbot larvae breeding hatchery management practices. The other nutrient additives used in the nutrient enrichment process were identical in the different treatments. The live feed was fed to the turbot larvae after filtration and washing.

### Experimental design

Turbot larvae were distributed in farming ponds with a capacity of 15 m^3^. The experimental group and control group were assigned to two randomly selected ponds and the same amounts of individual larvae were added to each pond. The larvae were fed on live feed with added *B*. *amyloliquefaciens* in the experimental group whereas live feed without the probiotic was used to feed the larvae in the control group from 3 to 27 days after hatching. Throughout the developmental stage, the turbot larvae were fed on *B*. *plicatilis* during days 3–13 and on *A*. *sinica* during days 10–27, where pelleted feed was gradually added after 16 days. The frequency of feeding with *B*. *plicatilis* or *A*. *sinica* was three times per day, the pelleted feed was added via automatic feeding machine once every few minutes. During days 16–23, the amount of *A*. *sinica* was about 1.1×10^8^ and the amount of pelleted feed was increased gradually from 24 g to 120 g. After adaptation to the pelleted feed, the amount of *A*. *sinica* was decreased gradually to 2.8×10^7^ on day 27 and the amount of pelleted feed was increased gradually to 396 g. The diameter of pelleted feed was 0.25–0.36 mm during days 16–26 and 0.36–0.58 mm after day 27. The amount of pelleted feed ingested changed over time according to the growth and decreased density of larvae. The breeding process strictly adhered to the practices employed in the turbot larvae breeding hatchery.

### Sampling of turbot larval intestinal tract

**Fig 1:**
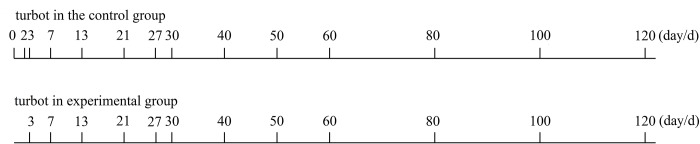
Sampling times for turbot larvae under the different treatments. The first feed for turbot larvae was started on day 3 and they fed on *B*. *plicatilis* processed in different treatments, whereas the samples on days 0 and 2 represented fertilized eggs and larvae without opening, respectively. Therefore, the first larvae samples in the experimental group were obtained on day 3.

As shown in Fig 1, larvae samples were collected at 9:00 am before the next feed throughout the test period. The larvae samples (days 2–120) were washed three times in sterile seawater for 20 min each time. After wiping with 75% alcohol, five randomly selected normal larvae sampled from the different treatments on each sampling day were dissected and the intestinal tract was removed with sterile scissors and forceps. The contents were then squeezed out and the intestinal tracts were stored after washing with sterile saline. The entire larva was retained if its length was quite small. After washing several times, fertilized eggs (0 day) was also distributed into 1.5 mL tubes, with 10 normal eggs in each tube.

### DNA extraction and sequencing

An E.Z.N.A. Soil DNA Kit (Soil DNA Kit D5625, Omega Biotek, USA) was used to extract the total DNA from each sample (intestinal tracts of turbot larvae, entire small turbot larvae, and fertilized eggs) according to the manufacturer’s instructions. The V3 and V4 regions of 16S rDNA were amplified by PCR using the following primers: 341F (5′-CCTAYGGGRBGCASCAG-3′) and 806R (3′-GGACTACNNGGGTATCTAAT-5′). After the amplified DNA was confirmed with agarose gel electrophoresis, high throughput sequencing was conducted with an Illumina HiSeq PE250 system.

### Data analysis

The raw data obtained from high throughput sequencing were processed by splitting, splicing, filtering, and extracting before obtaining effective tags. The total effective tags obtained from all of the samples were clustered using Uparse v7.0.1001 [33]. Several effective tags were clustered into operational taxonomic units (OTUs) when their shared sequencing identity was higher than 97%. Representative sequences of OTUs were selected and annotated in species using the RDP Classifier 2.2 [34] and Greengenes database [35] where the threshold was set at 0.8–1.0. Data for all samples were homogenized based on the standard for the sample with the least data. Alpha and beta diversity analyses were performed based on this homogenization process.

The ACE value and Shannon index of alpha diversity were calculated at the OTU level [36]. The Student’s *t*-test was used to detect significant differences between the experimental group and the control group. The significance level was set at *P* < 0.05 and the results were expressed as the mean ± the standard error of the mean.

### Example ethics statement

The samples used in this study were marine-cultured animals, and all of the experiments were strictly conducted according to the regulations from local government and the Institutional Animal Care & Use Committee (IACUC) of Yellow Sea Fisheries Research Institute, Chinese Academy of Fishery Sciences. All surgery was performed under MS-222 (Fluka, USA) anesthesia, and all efforts were made to minimize suffering.

## Results

### Statistics of sequencing data

**Fig 2:**
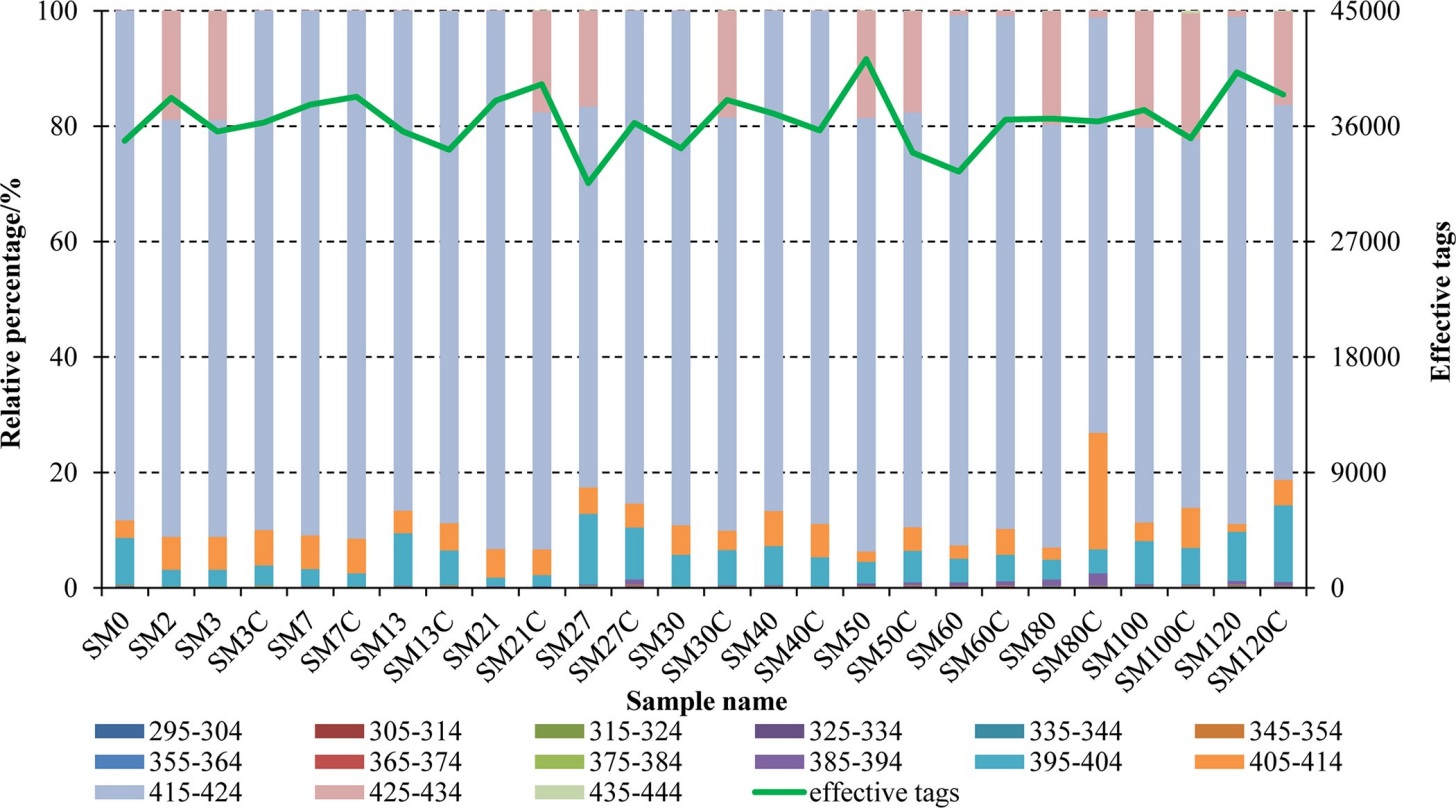
Length distributions of effective tags in different samples. For each sample, we calculated the average based on five replicates in the same distribution range. SM0 and SM2 represent fertilized eggs and turbot larvae on day 2, respectively. SM3, SM7, SM13, …, SM100, and SM120 represent turbot larval intestinal tract samples on days 3, 7, 13, …, 100, and 120 in the experimental group, respectively. Similarly, SM3C, SM7C, SM13C, …, SM100C, and SM120C represent turbot larval intestinal tract samples on days 3, 7, 13, …, 100, and 120 in the control group, respectively.

We removed the barcode and primer sequences, and cleaned the unqualified tags and chimera sequences for the raw data obtained from 126 samples using the Illumina HiSeq PE250 system. Thus, the effective tags were obtained for use in the subsequent analyses. Each sample had an average of 36,540 effective tags (Fig 2).

The length distribution of the total tags was mainly 415–424 bp according to the analysis (Fig 2). The relative proportion of the main length distribution was higher than 60% in all samples and the average was 80.61%. Moreover, the length distributions of the remaining total tags were 395–404 bp and 405–414 bp, with relative proportions of 1.5%–13.3% and 1.3%–20.2%, respectively. However, the length distribution of the tags was 425–434 bp in a few samples, i.e., 0–21.8%.

### Alpha diversity indexes

**Fig 3:**
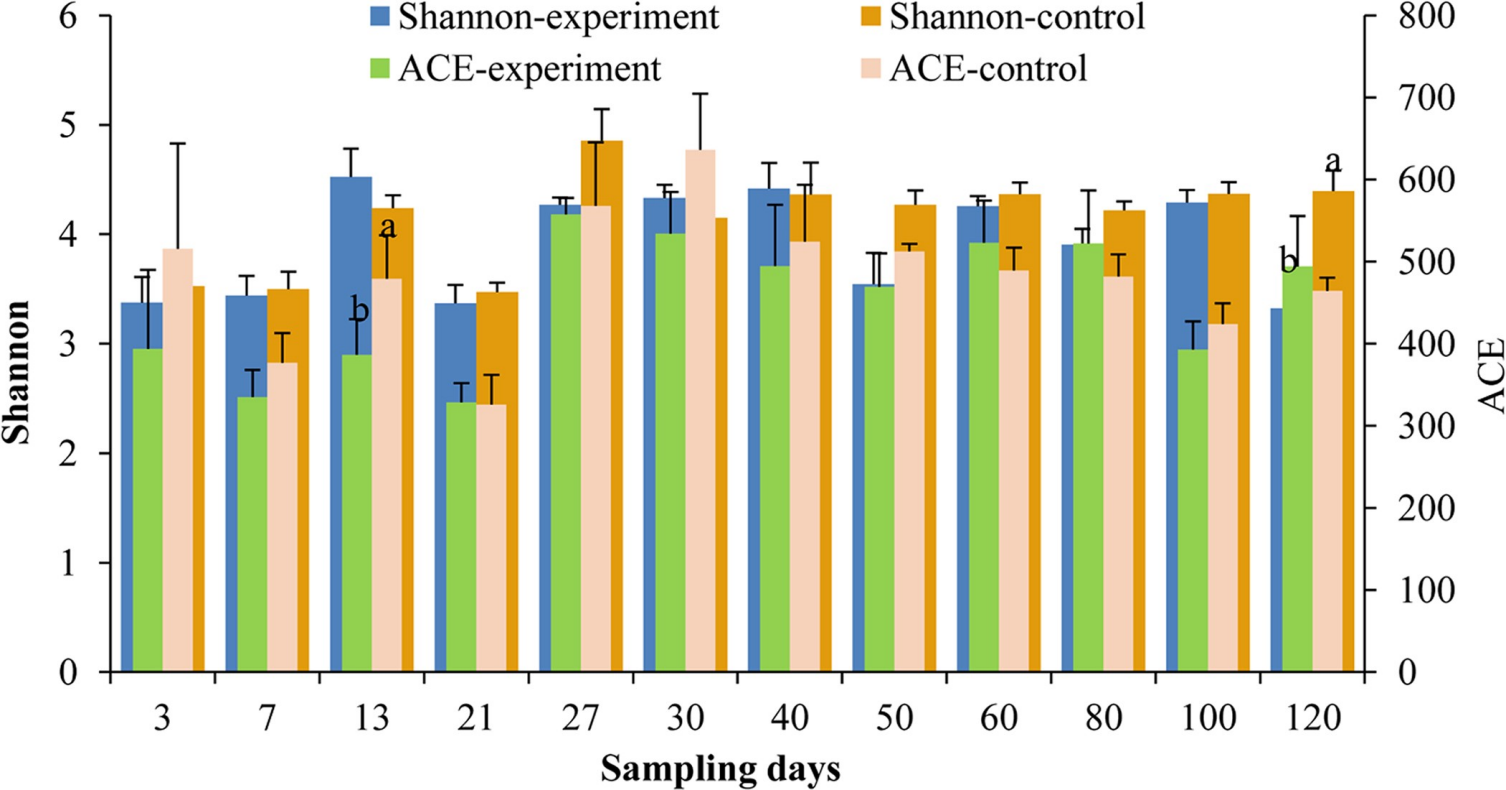
Abundance-based coverage estimator (ACE) and Shannon index for turbot larvae intestinal microflora during the developmental stage. The ACE or Shannon index for each sample was the average based on five replicates. Values with different letters differed significantly on the same day (*P* < 0.05).

The abundance-based coverage estimator (ACE) is a common index used to estimate the abundance of microbial species in ecology. Under different treatments from days 3 to 120, the ACE values increased initially and then decreased (Fig 3). Throughout the whole developmental period, the highest ACE value (557.3) was obtained on day 27 in the experimental group and on day 30 in the control group (636.4). In the stage with live feed during days 3–27, excluding day 21, the ACE values were lower in the experimental group than the control group, and there was a significant difference between treatments on day 13 (*P* < 0.05). Compared with the control group, the ACE values were lower in the experimental group when provided only with pelleted feed on days 30–120, excluding days 60, 80, and 120.

The diversity of the microbial community was expressed using the Shannon index. The changes in the Shannon index under different treatments during the turbot larvae developmental stage are shown in Fig 2. The Shannon indexes were lower during days 3–21 than days 27–120. The highest Shannon index (4.86) was determined on day 27 in the control group. Similarly, the Shannon index values were lower in the experimental group than the control group, except on days 13, 30, and 40 during the developmental stage. However, there was only a significant difference between the experimental group and the control group on day 120 (*P* < 0.05).

Therefore, the addition of *B*. *amyloliquefaciens* could reduce the abundance of microbial species and the diversity of the microbial community, and this effect might persist in later developmental stages.

### Microflora composition analysis

**Fig 4:**
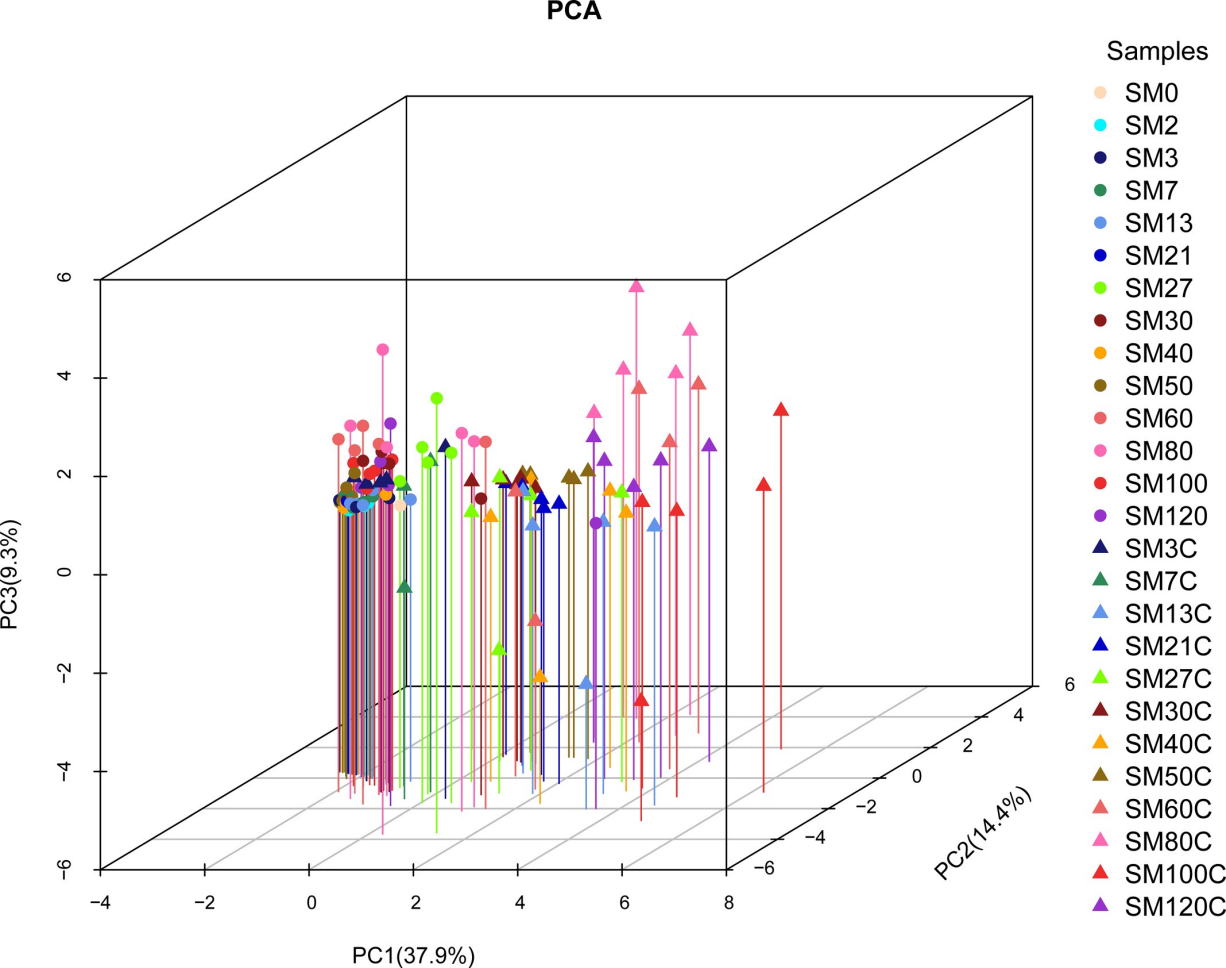
Principal components analysis (PCA) results based on the operational taxonomic units level in turbot larvae guts under different treatments.

The microflora structures in the turbot larvae intestines under different treatments are shown in Fig 4 based on the OTU level. PC1, PC2, and PC3 were the three main principal components according to principal components analysis, where PC1, PC2, and PC3 explained 37.9%, 14.4%, and 9.3% of the variation, respectively. The distance between two points indicates the similarity of the microbial community structure where each point represents a sample. We found that the distribution of the samples was highly dispersed in the control group. The distance between samples was small on days 0 and 2. On most other days, the samples were distributed far from the samples on days 0 and 2 in the control group. By contrast, all of the samples were distributed quite close together in the experimental group. Moreover, the distances between the samples in the experimental group were relatively small on days 0 and 2. Therefore, the addition of *B*. *amyloliquefaciens* changed the composition of the microflora in the turbot larvae intestines, where it helped to stabilize and homogenize the microbial communities. This effect on the microflora structure might also have persisted in the intestine during the later developmental stages in turbot larvae.

**Fig 5:**
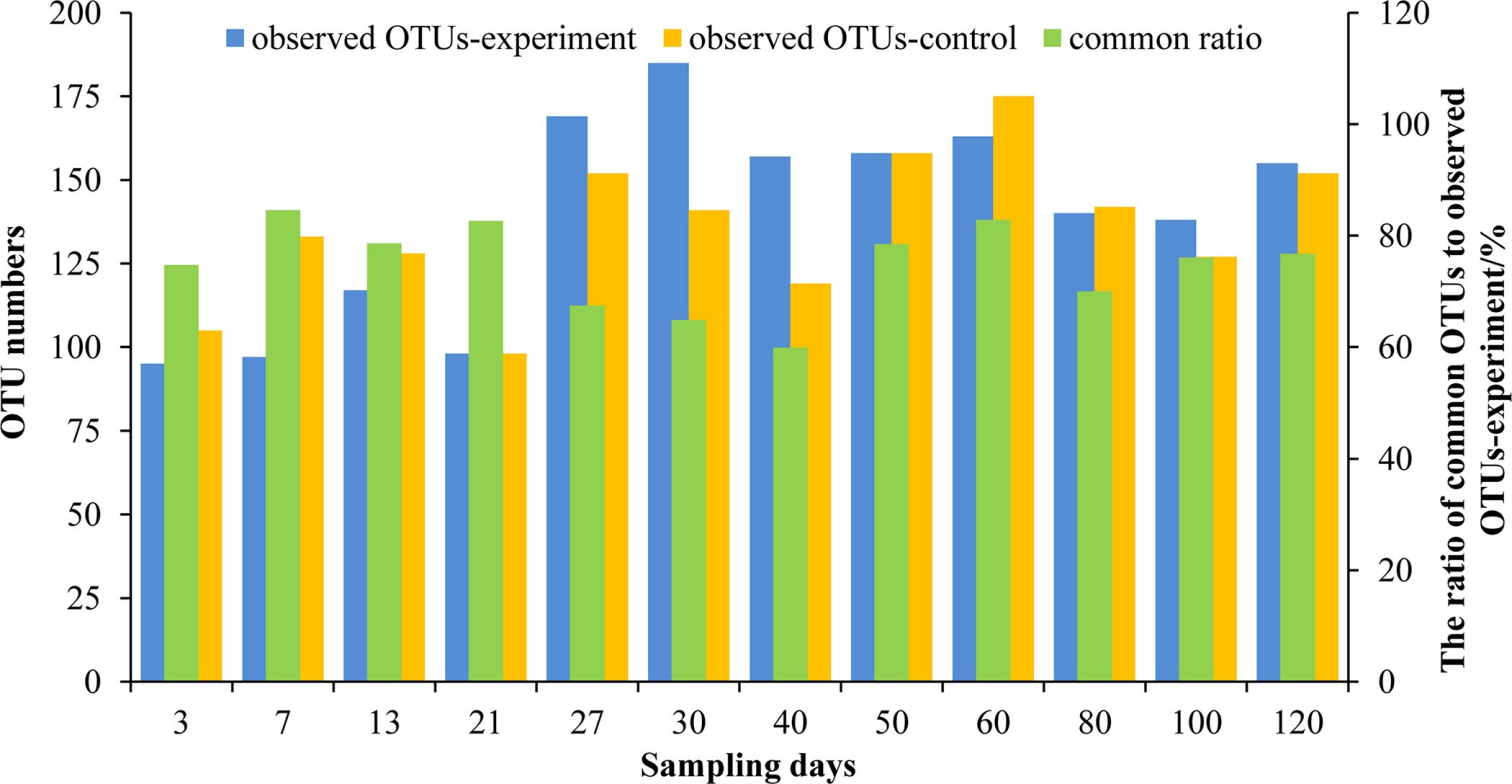
Similarity of the microflora in the intestinal tracts of turbot larvae under different treatments based on the operational taxonomic units (OTU) level. The OTUs in each sample were averaged based on five replicates.

The similarity of the microbial communities in the intestinal tract in the two groups was analyzed based on the OTUs during the growth of the turbot larvae (Fig 5). The changes in the number of OTUs in the experimental group were similar to those in the control group. The numbers of OTUs were lower in the experimental group than the control group on days 3–13 and 60–80, but higher on days 27–40 and 100–120, and equal on days 21 and 50. Moreover, the differences in the numbers of OTUs during days 3–40 were greater than those in the later stage between the two groups. The highest number of OTUs (185) was determined on day 30 in the experimental group. The proportion of common OTUs in both groups increased initially and then decreased. The highest proportion of common OTUs (84.5%) occurred on day 7 and the lowest (59.9%) on day 40. The average proportion of common OTUs was 74.7% in both treatments throughout the whole developmental period. Thus, the addition of *B*. *amyloliquefaciens* affected the total number of OTUs and the proportion of common OTUs in the intestines of turbot larvae.

### Changes of dominant microbial community

**Fig 6:**
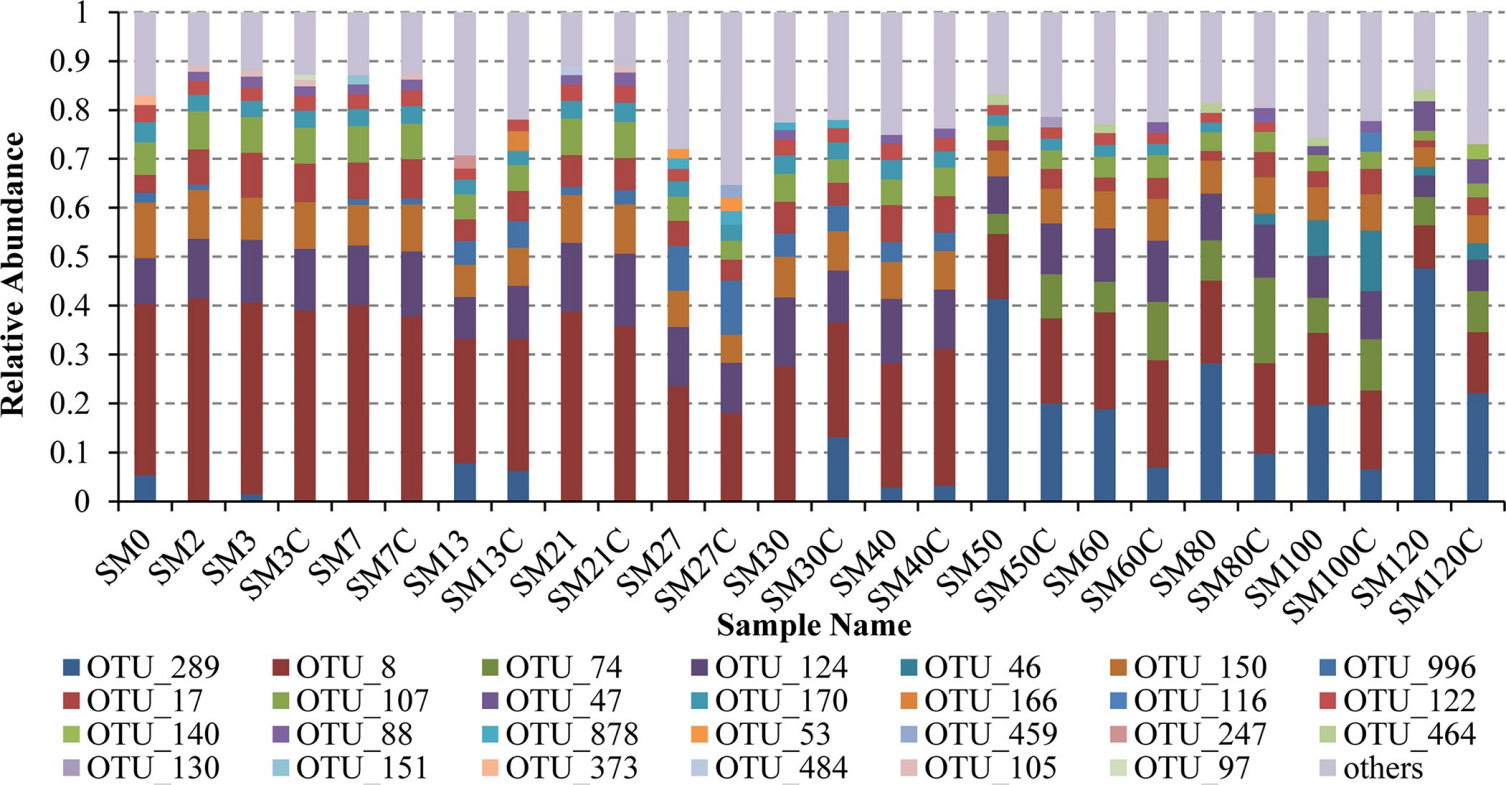
Microflora structure in the guts of turbot larvae under different treatments on each sampling day. For each sample, the averages were obtained based on five replicates. Each bar represents the top 10 operational taxonomic units (OTUs) in each sample.

The top 10 OTUs in each sample are shown in Fig 6, where all of the OTUs in the samples were averaged based on five replicates, except on day 0. Before the first feeding, microbial communities were present in the intestines of the turbot larvae on days 0 and 2. During days 0–40, the microflora structure was similar under the different treatments on each day, where the abundance of OTU8 was the highest, and the other major OTUs included OTU124, OTU150, OTU107, and OTU17. The summed relative abundance of these major OTUs was greater than 0.53 in each sample, except on day 27. During days 50–120, the relative abundance of OTU289 increased and it replaced OTU8 as the most abundant OTU. In addition, OTU289, OTU8, OTU74, OTU124, OTU150, OTU107, and OTU17 were dominant, where the summed abundances of these OTUs was higher than 0.60 in each sample. Hence, OTU8, OTU150, OTU124, OTU17, and OTU107 were always the common dominant OTUs in the intestines of turbot larvae throughout the whole developmental period. Moreover, the types and relative abundances of the dominant OTUs were similar in the intestinal tract, which indicates that the compositions of the major microflora were similar throughout the whole developmental period in the turbot larvae. During the developmental period, there were obvious changes in the abundances of OTU289 and OTU74, which were the main differences between the early and later stages. OTU8 was always dominant, although its relative abundance decreased. The changes in the relative abundances of these three major OTUs were distinct in the different treatments. During days 3–30, the abundance of OTU8 was always higher in the experimental group than the control group, but the trend was reversed during days 40–120. Compared with the control group, the relative abundance of OTU289 was higher in the experimental group on days 40–120. During days 50–120, the abundance of OTU74 increased and it became dominant, but the abundance of OTU74 was lower in the experimental group than the control group. Therefore, the introduction of *B*. *amyloliquefaciens* into the live feed affected the structure of the main microflora in the intestines of turbot larvae during the stage when live feed was provided. However, the effects on the main microbial components were more obvious in the stage with pelleted feed and without the addition of *B*. *amyloliquefaciens*. These results indicate that the effects of *B*. *amyloliquefaciens* on the major structure of the microflora in the intestinal tract were clearer in the later stage of development.

The annotation information of dominant OTUs shown in Fig 6 is presented in Table 1. At the phylum level, most of these OTUs belonged to Firmicutes and Proteobacteria, where Bacilli and Gammaproteobacteria were the major classes. At the genus level, *Lactococcus*, *Arthrobacter*, *Solibacillus*, and *Pseudomonas* were the major genera. OTU8 and OTU289 were highly abundant and they were classified as *Lactococcus* and Vibrionaceae. In addition, OTU8 and OTU107 were classified as *Lactococcus*, whereas OTU122 and OTU996 belonged to *Pseudomonas*.

**Table 1:** Dominant operational taxonomic units (OTUs) in the intestinal tracts of turbot larvae.

OTU number	The closest strain in GreenGene (Phylum/Class/Order/Family/Genus)
8	Firmicutes/Bacilli/Lactobacillales/Streptococcaceae/*Lactococcus*
107	Firmicutes/Bacilli/Lactobacillales/Streptococcaceae/*Lactococcus*
97	Firmicutes/Bacilli/Lactobacillales/Streptococcaceae/*Streptococcus*
151	Firmicutes/Bacilli/Lactobacillales/Lactobacillaceae/*Lactobacillus*
105	Firmicutes/Bacilli/Lactobacillales/Carnobacteriaceae/*Carnobacterium*
150	Firmicutes/Bacilli
124	Firmicutes/Bacilli/Bacillales/Planococcaceae/*Solibacillus*
88	Firmicutes/Bacilli/Exiguobacterales/Exiguobacteraceae/*Exiguobacterium*
289	Proteobacteria/Gammaproteobacteria/Vibrionales/Vibrionaceae
166	Proteobacteria/Gammaproteobacteria/Vibrionales/Vibrionaceae/*Aliivibrio*
170	Proteobacteria/Gammaproteobacteria/Pseudomonadales/Pseudomonadaceae
122	Proteobacteria/Gammaproteobacteria/Pseudomonadales/Pseudomonadaceae/ *Pseudomonas*
996	Proteobacteria/Gammaproteobacteria/Pseudomonadales/Pseudomonadaceae/ *Pseudomonas*
484	Proteobacteria/Gammaproteobacteria/Oceanospirillales/Halomonadaceae/*Cobetia*
74	Proteobacteri/Gammaproteobacteria/Oceanospirillales/Halomonadaceae
878	Proteobacteria/Gammaproteobacteria/Aeromonadales/Aeromonadaceae
459	Proteobacteria/Gammaproteobacteria/Alteromonadales/Pseudoalteromonadaceae/*Pseudoalteromonas*
53	Proteobacteria/Gammaproteobacteria/Enterobacteriales/Enterobacteriacea/ *Escherichia*
464	Proteobacteria/Gammaproteobacteria/Enterobacteriales/Enterobacteriaceae/ *Edwardsiella*
130	Proteobacteria/Alphaproteobacteria/Sphingomonadales
247	Proteobacteria/Alphaproteobacteria
17	Actinobacteria/Actinobacteria/Actinomycetales/Micrococcaceae/*Arthrobacter*
373	Cyanobacteria/Chloroplast/Streptophyta
140	Fusobacteria/Fusobacteria/Fusobacteriales/Fusobacteriaceae/*Psychrilyobacter*
116	Unclassified
46	-
47	-

Note: OTU46 and OTU47 were only classified to the kingdom of bacteria.

**Fig 7:**
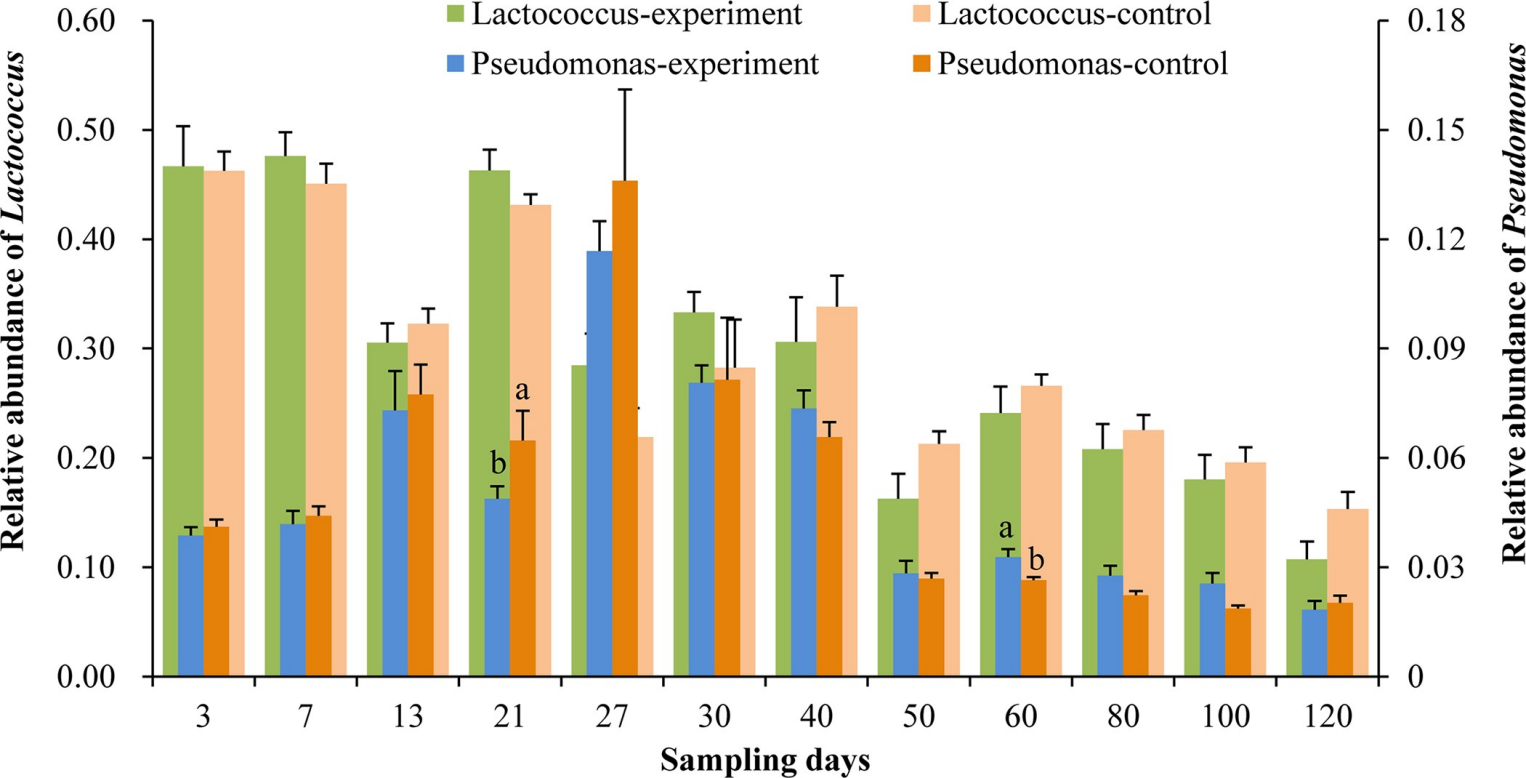
Relative abundances of *Lactococcus* and *Pseudomonas* spp. in the intestines of turbot larvae. For each sample, the averages were obtained based on five replicates. Values with different letters were significantly different on the same day (*P* < 0.05).

The relative abundances of all the OTUs that belonged to *Lactococcus* and *Pseudomonas* are compared in Fig 7. Excluding day 13, the abundances of *Lactococcus* spp. were higher in the experimental group than the control group, with 0.4%–6.6% during days 3–30 and the average value was 3.5%, but the abundances were lower during days 40–120. However, the abundances of *Pseudomonas* spp. were lower in the experimental group than the control group, with 0.08%–1.9% (mean = 0.8%) during days 3–30, but the abundances were higher during days 40–120. Moreover, there were significant differences on days 21 and 60 (*P* < 0.05). Thus, the introduction of *B*. *amyloliquefaciens* in the live feed reduced the abundances of *Pseudomonas* and increased the abundances of *Lactococcus* in the earlier stage during days 3–30, but this effect did not persist in the later developmental stage.

## Discussion

Previous studies have shown that Firmicutes, Proteobacteria, and Bacteroidetes are dominant communities in many kinds of fish at the phylum level [37–41]. Similar results were obtained in the present study, where Firmicutes and Proteobacteria were dominant, and they accounted for 60% of the tags at the phylum level in the microbial communities found in the guts of turbot larvae (Fig 4 and Table 1).

During days 0–40, the relative abundance of OTU8 was 0.18–0.41 (mean = 0.32), which was the highest in the different treatments. However, the average abundance of OTU289 was 0.22 (0.07–0.48) during days 50–120 whereas that of OTU8 was 0.16 (0.09–0.22). The dominant OTUs were OTU289 or OTU8 in the later stage. OTU8 and OTU289 were classified as belonging to *Lactococcus* and Vibrionaceae, respectively. In addition, OTU107 was highly abundant and it was classified as *Lactococcus* according to the phylogenetic analysis. The relative abundance of *Lactococcus* was 0.11–0.49 (mean = 0.31). Therefore, *Lactococcus* was the most dominant genus in the intestines of healthy turbot larvae throughout the entire developmental period (Figs 3 and 7, Table 1). *Lactococcus* includes species such as *L*. *garvieae*, *L*. *lactis* (including *L*. *lactis* subsp. lactis, *L*. *lactis* subsp. *cremoris*, and *L*. *lactis* subsp. *hordniae*), *L*. *raffinolactis*, *L*. *piscium*, and *L*. *plantarum* as five clearly separated species [42], while *L*. *chungangensis* [43] and *L*. *fujiensis* [44] are two newly described species. *Lactococcus* species are nutritionally fastidious and they require a complex medium to achieve their best growth [45, 46], such as Elliker medium [47] and M17 medium [48]. *L*. *lactis* is used widely as a starter culture in fermented dairy products [49]. Some subspecies such as *L*. *lactis* subsp. lactis can produce many different antibacterial substances, including nisin, which strongly reduces the survivability of undesirable Gram-positive bacteria in a wide range of conditions [50, 51]. *L*. *garvieae* was first reported as a pathogen in farmed trout at the end of the 1950s in Japan [52], and it was later reported in freshwater prawn (*Macrobrachium rosenbergii*) [53] and grey mullet (*Mugil cephalus*) [54]. *L*. *piscium* was first isolated from a diseased rainbow trout [55] but there is no direct evidence of its pathogenicity. Thus, no previous reports have demonstrated that any diseases are caused by *Lactococcus* in turbot. In the present study, the normal and healthy turbot larvae had high abundances of *Lactococcus* in their intestines, but they did not exhibit any disease symptoms throughout the developmental period, thereby indicating a low abundance of pathogenic species or none in the genus *Lactococcus*. The composition and roles of *Lactococcus* in the intestinal tracts of turbot should be investigated in future studies.

Higher relative abundances of *Lactococcus* were observed in the experimental group during the earlier stage from days 3–30, but not on day 13. Thus, the addition of *B*. *amyloliquefaciens* might have improved the adhesive or reproductive capacity of *Lactococcus* in the larval intestinal tract, although this effect disappeared on days 40–120. The turbot larvae were fed on *B*. *plicatilis* from the first feeding until 13 days, and the microbial community structure gradually tended to stabilize. *A*. *sinica* containing different microbes compared with *B*. *plicatilis* was added to the diet on day 10 and feeding with *B*. *plicatilis* was ended on day 13. The response of the microbial community in the gut to this change might explain why the relative abundance of *Lactococcus* was lower in the experimental group than the control group, as well as the fluctuations in the ACE and Shannon index values, the changes in the composition of the top 10 OTUs, and the proportion of shared OTUs on day 13. The pelleted feed was added gradually on day 16 and the amount of *A*. *sinica* was reduced. The size and the amount of pelleted feed changed gradually with the growth of the turbot larvae. Thus, the microbial community in the intestine changed on days 21 and 27, as indicated by the changes in the ACE value, total abundance of the top 10 OTUs, and the proportion of shared OTUs, thereby suggesting that the structure of the microflora in the intestine varied according to the size of the larvae and their diet. Similarly, some previous studies have indicated that the structure of the intestinal bacterial community might be affected directly by dietary components [56–58]. In addition, due to the growth of the fish larvae in aquaculture, the intestinal system might provide more niches for bacteria, which could affect the microbial composition of the intestine [59, 60].

The changes in OTU8, OTU289, and OTU74 as the dominant OTUs, the structure of the microflora in the intestine, and the principal components analysis results demonstrated that the introduction of *B*. *amyloliquefaciens* in the live feed during nutrient enrichment modified and stabilized the composition of the microbial community in the intestine. This influence might also have affected the microflora structure in the intestine during the later stages of development. A previous study suggested that the addition of a probiotic in live feed affected the microflora structure in the intestine of the Atlantic halibut (*Hippoglossus hippoglossus*) [61]. By contrast, Bakke et al. [62] observed that the bacteria present in live feed had little effect on the microbial community in the intestines of fish larvae. This different results might be associated with the number of days considered in the experiments as well as the size and species of fish [63–66].

*Pseudomonas* is considered as potentially pathogenic bacteria in marine cultured fish [67, 3] such as turbot [68, 69] and cod [70]. However, no disease symptoms were detected during our experiment. Moreover, the abundances of *Pseudomonas* spp. were lower in the experimental group than the control group during days 3–30, whereas the opposite trend was found in the later stage, thereby demonstrating that the addition of *B*. *amyloliquefaciens* could actively inhibit the growth and reproduction of bacteria in the intestines of turbot larvae. However, this effect was not observed in the later stage.

*B*. *amyloliquefaciens* originated from the guts of farmed healthy and normal turbot, and it was added to the live feed [18, 62] ingested by the turbot larvae in the experiment. However, no specific primers were available to distinguish *B*. *amyloliquefaciens* from *Bacillus subtilis*, so the quantities of *B*. *amyloliquefaciens* could not be detected by real-time Polymerase Chain Reaction (RT-PCR). Furthermore, the OTUs representing the genus *Bacillus* were not dominant in the live feed [71] as well as in guts of the turbot larvae and juveniles. Hence, we inferred that the abundance of *B*. *amyloliquefaciens* was rather low in live feed as well as in guts of the turbot larvae and juveniles. However, the structure of the microbiota in live feed was clearly changed by the addition of *B*. *amyloliquefaciens* [71]. Similarly, the structures of microbiota in turbot larval intestines were also influenced. Our findings indicate that the live feed can transit the effect of *B*. *amyloliquefaciens* to the turbot larvae. This effect produced by *B*. *amyloliquefaciens* might inhibit the growth and reproduction of some bacteria (e.g., *Pseudomonas*) by competing for nutrition and adhesion sites, where the growth and reproduction of other microflora (e.g., *Lactococcus*) were improved via mutual benefits, thereby indicating the value of *B*. *amyloliquefaciens* as a probiotic. Skjermo et al. [72] used live feed and rearing water as the main routes for introducing bacteria originating from cod larvae into the intestines of other cod larvae, but the added bacteria were not able to colonize and persist in the intestinal tract during any of the developmental stages.
